# The Effect of Romantic Relationships on the Evaluation of the Attractiveness of One’s Own Face

**DOI:** 10.1177/2041669518765542

**Published:** 2018-04-17

**Authors:** Jiaye Cai, Yan Zheng, Pei Li, Bin Ye, Hongyan Liu, Liezhong Ge

**Affiliations:** Department of Psychology, 12646 Zhejiang Sci-Tech University, Hangzhou, China; Center for Psychological Sciences, Zhejiang University, Hangzhou, China

**Keywords:** romantic relationship, attractiveness evaluation, self-face, probability evaluation, subjective rating

## Abstract

The present study sought to explore the effect of romantic relationships on the attractiveness evaluation of one’s own face using two experiments with the probability evaluation and the subjective rating method. Experiment 1 and Experiment 2 enrolled couples and single individuals as participants, respectively. The results of the two experiments indicated that the participants evaluated their own face as significantly more attractive than did others of the same sex. More importantly, the romantic relationship enhanced the positive bias in the evaluation of self-face attractiveness, that is, couple participants showed a stronger positive bias than did single individuals. It was also found that a person in a romantic relationship was prone to overestimating the attractiveness of his or her lover’s face, from the perspective of both probability evaluation and rating score. However, the abovementioned overestimation did not surpass the evaluations of the exaggeratedly attractive face. The present results supported the observer hypothesis, demonstrating the romantic relationship to be an important influential factor of facial attractiveness. Our findings have important implications for the research of self-face evaluation.

## Introduction

Facial attractiveness is a type of positive experience evoked by the target’s face that induces preferences (Rhodes, 2006). Facial attractiveness embodies a great deal of social affective information, such as age, gender, social status and emotion, and it plays an important role in social interaction.

From the 1970s to the present, numerous studies have investigated what renders a face attractive. Research primarily focused on the averageness, symmetry and sexual dimorphism of faces. Converging evidence suggested that faces with high averageness, symmetrical faces and faces with sexual dimorphism (i.e. masculinity in male faces and femininity in female faces, which signal sexual maturity and reproductive potential) were more attractive (see Rhodes, 2006, for a review). In addition, some other characteristics of faces were also addressed, such as the feature size of eyes, mouth, nose, chin, cheekbones, facial skin health and skin colour.

The aforementioned study investigated facial attractiveness from the perspective of other people. Some studies also addressed the self-evaluations of facial attractiveness. The first such study came from Cash, Cash, and Butters (1983). In their study, female participants were requested to rate their own attractiveness following exposure to same-sex persons who were either physically attractive or unattractive. The results indicated that subjects gave lower self-ratings following the attractive compared with the less attractive stimulus contrast. The findings supported the contrast effect and were consistent with social comparison theory. Some other studies obtained similar results and supported the theory (e.g. Little & Mannion, 2006; Thornton & Maurice, 1999). However, these investigations primarily explored the influence of context (e.g. social comparison stimulus) on self-evaluations of facial attractiveness.

Several recent studies investigated the self-evaluations of facial attractiveness compared with evaluations of their facial attractiveness by others. In a study conducted by Springer et al. (2012), subjects were required to evaluate four photos of themselves using a variety of descriptive sentences, such as the statement ‘I look very young’. The results indicated that compared with others’ evaluation, people evaluated their own faces as more attractive. In our recent study (Cai, Zheng, Ge, & Liu, 2018), we observed similar results using a more objective method of paired comparison. A study by Stillman and Fristina (2010) also determined that female patients in a dermatology office rated themselves as more attractive than did other persons who viewed photographs of those patients. Such studies consistently conclude that individuals maintain a positive bias in evaluating self-face attractiveness.

Existing findings suggest that a relationship, particularly romantic love, may affect the self-evaluation of facial attractiveness. For example, numerous studies have indicated a positive illusion called the ‘love-is-blind bias’, which refers to one’s evaluation of a lover’s physical attractiveness as being higher than one’s evaluation of one’s own attractiveness (Barelds-Dijkstra & Barelds, 2008; Hall & Taylor, 1976; McClanahan & Shafir, 1990; Swami, Stieger, Haubner, Voracek, & Furnham, 2009), even in homosexual relationships (Swami et al., 2009). These studies emphasize the influence of the romantic relationship on the evaluation of the lover’s facial attractiveness. Because romantic love is an interactive relationship, both parties not only change their assessment of the lover’s attractiveness but may also change their assessment of self-face attractiveness. In addition, the romantic relationship is highly associated with self-esteem (Bylsma, Cozzarelli, & Sumer, 1997), and self-esteem was observed to relate to self-evaluation of facial attractiveness (Bale, 2010). Thus, the romantic relationship may affect the self-evaluation of facial attractiveness. However, to our knowledge, no study to date has explored this issue directly.

The present study seeks to examine the influence of a romantic relationship on the evaluation of self-face attractiveness. We predicted that romantic love enhances the positive bias in the evaluation of self-face attractiveness, that is, individuals in love will evaluate their facial attractiveness to be higher than individuals in a single state. For this purpose, two experiments were conducted. Experiment 1 recruited individuals who were falling in love as subjects to investigate the characteristics of the self-evaluation of facial attractiveness compared with others’ and the lover’s evaluation. Experiment 2 examined single subjects as a contrast group to the couple participants in Experiment 1. Furthermore, exaggeratedly attractive faces that represented a so-called perfect face with extremely high attractiveness (Perrett, May, & Yoshikawa, 1994; Rhodes & Tremewan, 1996; Yu, Ge, Sun, & Wang, 2013) were introduced to examine the extent of the overestimation of self-face attractiveness. In the current study, two evaluation methods were adopted, that is, a subjective rating measurement and a relatively objective method of paired comparison in which two faces were paired and the evaluators were asked to judge which one was more attractive.

## Experiment 1: The Evaluation of Self-Face Attractiveness in Individuals in Romantic Relationships

Experiment 1 recruited couples as participants to investigate the characteristics of the evaluation of self-face attractiveness. In addition, the experiment explored whether one’s lover would overestimate the attractiveness of the partner’s face.

### Methods

#### Participants

A total of 30 heterosexual couples (i.e. 60 participants; mean age 20.7 ± 1.5 years with a range of 18–24 years) participated in this experiment for monetary compensation. All were right-handed native Mandarin Chinese speakers with normal or corrected-to-normal vision. All of the participants were required to complete the Chinese adaptation of the ECR (Experiences in Close Relationships) Inventory (Li & Kato, 2006) to ensure that all of the couples were in stable public relationships with lovers. By asking each participant whether they knew the other participants, we verified that none of the couples knew one another before the experiment. Written informed consent was obtained from each participant following a research protocol approved by the Institutional Review Board of Zhejiang Sci-Tech University. All experimental methods were conducted in accordance with the approved guidelines regarding all relevant aspects, including the recruitment, experimental process information, compensation and debriefing of participants.

#### Materials

*Self-face images*

One standardized image was obtained for each participant before the formal experiment following several steps. First, a full-face photograph was taken at a specified distance (1.5 m) using a digital camera (model: FinePix S9600). The participants were required to tie back their hair to expose their entire face. In addition, they were asked to gaze directly at the camera with a neutral expression by referring to a standard neutral face. Two bright lights were focused on the left and right sides of the participant’s face to ensure sufficient and consistent light. Second, the original images were modified with the Adobe Photoshop software (http://www.adobe.com/products/photoshop.html): (a) to remove evident spots on the photos while keeping the face intact, (b) to adjust the image’s direction based on the horizon linking the two eyes, (c) to convert the colour photos to black-and-white photos and (d) to resize the images to 400 × 400 pixels. The final images consisted of an oval-shaped face on a black background.

#### Exaggeratedly attractive images

To find exaggeratedly attractive images, we first searched attractive faces on the Internet using keywords related to high attractiveness, such as ‘beauty’ and ‘handsome’. In this manner, we collected 125 photos (70 females) with a front neutral face on a white background. Then, the photos were processed to standardized images using the same steps used for the self-face images. Third, these faces were rated on a 7-point Likert scale (1 = *not at all attractive* to 7 = *most attractive*) by 61 college students (30 females, mean age 20.8 ± 1.8 years) who were not the formal subjects of the two experiments. Finally, we selected the female and male faces with the highest attractiveness scores (5.89 and 5.57, respectively) as the exaggeratedly attractive female and male faces.

#### Procedure

The formal experiment was conducted on two successive days. On the first day, female and male subjects completed the experiment separately, with each participant participating only in the evaluation of same-sex faces. The experiment was conducted in two stages. In the first stage, the paired comparison method was used. Two of the 31 same-sex facial images (30 same-sex subjects’ faces and the same-sex exaggerated attractiveness face) were paired and presented twice (one on the left side and one on the right side), resulting in a total of 930 pairs $\left(P\frac{2}{31}\right)$. For each trial, a ‘+’ was presented for 500 ms as a focal point, followed by one image pair. The participants were instructed to judge which of the two paired faces was more attractive. If they thought the left face was more attractive, then they pressed the ‘left’ button (the ‘F’ key on the keyboard). If they thought the right face was more attractive, they pressed the ‘right’ button (the ‘J’ key on the keyboard). The image pair did not disappear until a response was entered. After the response, the next trial began. The participants were exposed to a short practice session before the three formal sessions, and there was a short break of no less than 2 min during two successive sessions. After the first stage, there was a rest for approximately 2 min. In the second stage, the subjective rating method was used. For each trial, a ‘+’ was presented for 500 ms, followed by one facial image randomly selected from the 31 same-sex images (30 images of the same-sex subjects and the same-sex exaggeratedly attractive face). The participants were instructed to evaluate the attractiveness of the faces in the images based on a 7-point Likert scale (1 = *not at all attractive*; 7 = *most attractive*) by pressing one of the ‘1–7’ keys on the keyboard. After the response, the next trial began immediately. Participants also completed a short practice session before the formal session.

On the second day, the experimental procedure was the same as on the first day, except that the participants were asked to judge and evaluate the attractiveness of opposite-sex faces, including their lover’s face, faces of other opposite-sex subjects, and the opposite-sex exaggeratedly attractive face.

#### Data analysis

Two types of data were obtained in the experiment: the probability data (which indicates the probability of one’s own face being selected as more attractive when paired with other same-sex faces) and the rating scores data (which indicate the attractiveness rating scores of one’s own face). Thus, the following two sections introduced how the data were defined and calculated in different conditions.

*(1) Probability analysis*

In the present study, the probability (%) was defined as the ratio of selection, that is, the actual number of selection/the total number of presentations.

There were two types of pairing: one’s own face paired with the face of another same-sex subject (i.e. Self vs. Other) and one’s own face paired with the same-sex exaggeratedly attractive face (i.e. Self vs. Exaggerated). Thus, the probability data comprised the corresponding two types.

*(i) Probability of one’s own face being selected as more attractive when paired with other same-sex faces*

There were 30 faces of same-sex subjects in Experiment 1. A particular face could be paired with 29 other faces, thus resulting in 58 pairs (since each face could be placed either in the left or in the right position). Each pair could be evaluated by 30 same-sex subjects and 30 opposite-sex subjects.

First, we introduced a method to calculate the probability when the evaluator was the same sex. For a given image, if the evaluator was the subject of the image, then the probability of this image being selected as more attractive was referred to as ‘probability of one’s own face being selected as more attractive by oneself’ (the *Self* condition in Table 1); otherwise, the probability was referred to as ‘probability of one’s own face being selected as more attractive by other same-sex evaluators’ (the *ssOthers* condition in Table 1). For each image, there was one data point for the ‘Self’ condition and 29 data points for the ‘ssOthers’ condition. The final probability of the ‘ssOthers’ condition for each image was defined as the average of these 29 data points. The overestimation probability that one’s own image would be selected as more attractive was calculated by subtracting the probability of the ‘ssOthers’ condition from the probability of the ‘Self’ condition.

**Table 1.** Mean Probability (*SD*) of One’s Own Face Being Selected as More Attractive by Different Evaluators.

	Experiment 1						Experiment 2		
		Self vs. other			Self vs. exaggerated		Self vs. other		Self vs. exaggerated
	Self	ssOthers	Lover	osOthers	Self	Lover	Self	ssOthers	Self
Female faces	0.84 (0.14)	0.49 (0.15)	0.86 (0.11)	0.49 (0.12)	0.15 (0.33)	0.35 (0.32)	0.76 (0.21)	0.49 (0.17)	0.18 (0.30)
Male faces	0.90 (0.12)	0.49 (0.12)	0.87 (0.13)	0.49 (0.12)	0.30 (0.34)	0.37 (0.36)	0.86 (0.14)	0.49 (0.14)	0.30 (0.33)

*Note*. ssOthers = same-sex other evaluators; osOthers = opposite-sex other evaluators. ‘Self vs. Other’ is the probability of one’s own face being selected as more attractive when paired with other faces of the same-sex. ‘Self vs. Exaggerated’ is the probability of the self-face being selected as more attractive when paired with an exaggeratedly attractive face.

Next, we introduced a method to calculate the same probability when the evaluator was the opposite sex. For a given image, if the evaluator was the lover of the person in the image, then the probability of this image being selected as more attractive was referred to as ‘the probability of one’s own face being selected as more attractive by one’s lover’ (the *Lover* condition in Table 1); otherwise, the probability was referred to as ‘the probability of one’s own face being selected as more attractive by other opposite-sex evaluators’ (the *osOthers* condition in Table 1). For each image, there was one data point for the ‘Lover’ condition and 29 data points for the ‘osOthers’ condition. The final probability of the ‘osOthers’ condition was defined as the average of these 29 data points. The overestimation probability that each image would be selected as more attractive by one’s lover was calculated by subtracting the probability of the ‘osOthers’ condition from the probability of the ‘Lover’ condition.

*(ii) Probability of one’s own face being selected as more attractive when paired with the same-sex exaggeratedly attractive face*

A particular face could be paired with one same-sex exaggeratedly attractive face, thus resulting in two pairs. For a given image, if the evaluator was the subject of the image, then the probability of this image’s being selected as more attractive was referred to as ‘probability of selecting one’s own face as more attractive than the exaggerated image’ (the *Self* condition in Table 1). If the evaluator was the lover of the person in the image, then the probability of this image being selected as more attractive was referred to as ‘probability of one’s own face being selected as more attractive than the exaggerated image by one’s lover’ (the *Lover* condition in Table 1).

*(2) Rating scores analysis*
*(i) Attractiveness scores of self-face evaluation by different types of raters*

The subjective attractiveness evaluation for each image was measured by the rating scores based on a 7-point Likert scale. Each image could be evaluated by 30 same-sex subjects and 30 opposite-sex subjects.

First, we introduced the situation in which the rater was the same sex. For a given image, if the rater was the subject of the image, then the rating score for the image was referred to as ‘rating score by oneself’ (the *Self* condition in Table 2); otherwise, the rating score for the image was referred to as ‘rating score by other same-sex raters’ (the *ssOthers* condition in Table 2). For each image, there was one data point for the ‘Self’ condition and 29 data points for the ‘ssOthers’ condition. The final score of the ‘ssOthers’ condition for each image was defined as the average of these 29 data points. The overestimation score that each image would be selected as more attractive by oneself was calculated by subtracting the score of the ‘ssOthers’ condition from the score of the ‘Self’ condition.

**Table 2.** Mean Score (*SD*) of Attractiveness Rating of One’s Own Face and the Exaggerated Face by Different Raters.

	Experiment 1						Experiment 2		
	Self				Exaggerated		Self		Exaggerated
	Self	ssOthers	Lover	osOthers	Self	Lover	Self	ssOthers	Self
Female faces	4.6 (1.6)	3.3 (0.8)	5.7 (1.5)	2.9 (0.7)	6.3 (1.5)	6.7 (0.8)	4.0 (1.2)	3.3 (0.7)	6.3 (1.3)
Male faces	5.4 (1.8)	3.1 (0.5)	6.0 (1.9)	2.8 (0.6)	6.5 (1.0)	6.1 (1.8)	4.9 (1.8)	3.6 (0.8)	6.2 (1.4)

*Note*. ssOthers = same-sex other raters; osOthers = opposite-sex other raters.

Next, we introduced the situation in which the rater was the opposite sex. For a given image, if the rater was the lover of the person in the image, then the rating score for the image was referred to as ‘rating score by one’s lover’ (the *Lover* condition in Table 2); otherwise, the rating score for the image was referred to as ‘rating score by other opposite-sex raters’ (the *osOthers* condition in Table 2). For each image, there was one data point for the ‘Lover’ condition and 29 data points for the ‘osOthers’ condition. The final score of the ‘osOthers’ condition for each image was defined as the average of these 29 data points. The overestimation score that each image would be selected as more attractive by one’s lover was calculated by subtracting the score of the ‘osOthers’ condition from the score of the ‘Lover’ condition.

*(ii) Attractiveness scores of the exaggeratedly attractive face by different types of raters*

The rating score for the exaggerated image rated by the owner of the self-face image was referred to as ‘the rating score for exaggerated face by oneself’ (the *Self* condition in Table 2). The rating score for the exaggerated image rated by the lover of the person in the self-face image was referred to as the ‘rating score for exaggerated face by one’s lover’ (the *Lover* condition in Table 2).

### Results

The results of the experiments were presented in Tables 1 and 2.

#### Results of the probability evaluation

*(1) Results of the probability evaluation of one’s own face being selected as more attractive when paired with other same-sex faces (Self vs. Other)*

*(i) Results evaluated by same-sex subjects*

A Wilcoxon rank-sum test indicated that for both female (*Z* = −4.76, *p* < .001) and male (*Z* = −4.78, *p* < .001) faces, the probability of one’s own face being selected as more attractive by oneself (i.e. the *Self* condition in Table 1) was significantly higher than the probability that others of the same-sex would select that face (i.e. the *ssOthers* condition in Table 1). This result verified the positive bias in the evaluation of self-face attractiveness in couple participants from the perspective of probability evaluation.

*(ii) Results evaluated by opposite-sex subjects*

A Wilcoxon rank-sum test indicated that for both female (*Z* = −4.78, *p* < .001) and male (*Z* = −4.76, *p* < .001) faces, the probability of one’s own face being selected as more attractive by one’s lover (i.e. the *Lover* condition in Table 1) was significantly higher than the probability that one’s face would be selected by other opposite-sex participants (i.e. the *osOthers* condition in Table 1). This result revealed that the participants in love (compared with the others) were prone to overestimating the attractiveness of their lovers’ faces from the perspective of probability evaluation.

*(iii) Overestimation of the probability by oneself and the lover*

As described in the ‘Data analysis’ section, the overestimation probability of selecting one’s own face as more attractive was defined by the probability of ‘Self – ssOthers’, and the overestimation probability of being selected by one’s lover was defined by the probability of ‘Lover – osOthers’. The results of paired-samples *t* tests indicated that for both female face, *t*(29) = 0.94, *p* = .35, and male face, *t*(29) = −1.20, *p* = .24, the overestimation probabilities by oneself and by one’s lover were not significantly different.

1. *Results of probability evaluation of self-face selection as more attractive when paired with the same-sex exaggeratedly attractive face (Self vs. Exaggerated)*

*(i) Results evaluated by oneself*

A Wilcoxon rank-sum test was conducted to compare the mean probability of self-face selection as more attractive when paired with other same-sex faces (‘Self vs. Other’) with that when paired with the same-sex exaggerated face (Self vs. Exaggerated). The results indicated that for both female face (*Z* = −4.64, *p* < .001) and male face (*Z* = −4.66, *p* < .001), the probability of selecting one’s own face as more attractive in the ‘Self vs. Other’ condition was significantly higher than in the ‘Self vs. Exaggerated’ condition (see Table 1).

To determine whether a participant would think that his or her own face was more attractive than the exaggerated face, a one-sample *t* test was conducted with a random probability of 50%. The results indicated that for both the female, *t*(29) = −5.89, *p* < .001, and male, *t*(29) = −3.24, *p* < .01, faces, the probability of evaluating self-face attractiveness as higher than the exaggerated face was significantly lower than 50%. These results indicated that the positive bias in self-face attractiveness evaluation did not surpass the exaggerated faces in couples from the perspective of probability evaluation.

*(ii) Results evaluated by one’s lover*

A Wilcoxon rank-sum test was conducted to compare the mean probability of the lover selecting the partner’s face as more attractive when paired with other same-sex faces than when paired with the same-sex exaggerated face. The results indicated that for both female (*Z* = −4.52, *p* < .001) and male (*Z* = −4.48, *p* < .001) faces, the probability of the lover selecting the partner’s face as more attractive in the ‘Self vs. Other’ condition was significantly higher than in the ‘Self vs. Exaggerated’ condition (see Table 1).

To determine whether the participants’ lover would select the partner’s face as more attractive than the exaggerated face, a one-sample *t* test was conducted with a random probability of 50%. The results indicated that for the female faces, the probability of evaluating the attractiveness of partner’s face as higher than the exaggerated face was significantly lower than 50%, *t*(29) = −2.52, *p* < .05. By contrast, for male faces, there was no significant difference between the probability of evaluating the attractiveness of partner’s face as higher than the exaggerated face and 50%, *t*(29) = −1.98, *p* = .058.

#### Results of rating scores

*(1) Results of rating scores of self-face attractiveness*

*(i) Results rated by same-sex subjects*

Paired-samples *t* tests indicated that for both female face, *t*(29) = 3.69, *p* < .01, and male face, *t*(29) = 6.29, *p* < .001, the subjective rating score of attractiveness by oneself (i.e. the *Self* condition in Table 2) was significantly higher than the score by others of the same sex (i.e. the *ssOthers* condition in Table 2). This result also verified the positive bias in the evaluation of self-face attractiveness in couple participants from the perspective of rating score.

To investigate whether there was a gender difference in the attractiveness overestimation of one’s own face, we calculated the rating score differences between oneself and others of the same sex (i.e. ‘Self – ssOthers’) for both female (*M* = 1.2, *SD* = 1.8) and male (*M* = 2.3, *SD* = 2.0) faces. An independent-sample *t* test indicated that among those in close relationships, the degree of overestimation of males was much higher than that of the females, *t*(58) = 2.11, *p* < .05.

*(ii) Results rated by opposite-sex subjects*

Paired-samples *t* tests indicated that for both female face, *t*(29) = 10.66, *p* < .001, and male face, *t*(29) = 8.44, *p* < .001, the attractiveness rating score of one’s face by one’s lover (i.e. the *Lover* condition in Table 2) was significantly higher than the score by the opposite-sex others (i.e. the *osOthers* condition in Table 2). This result revealed that the participants in love were more prone to overestimating the attractiveness of their partners’ faces from the perspective of rating score.

To investigate whether there was a gender difference in the attractiveness overestimation of the lover’s face, we calculated the rating score differences between the lover and others of the opposite sex (i.e. ‘Lover – osOthers’) for both female (*M* = 2.8, *SD* = 1.4) and male (*M* = 3.2, *SD* = 2.1) faces. An independent-samples *t* test indicated no significant difference between the female and male faces, *t*(58) = −0.94, *p* = .36.

*(iii) Overestimation of the rating scores by oneself and the lover*

To directly compare one’s own attractiveness overestimation (i.e. ‘Self – ssOthers’) with that of one’s lover (i.e. ‘Lover – osOthers’), paired-samples *t* tests were conducted for female and male faces. The results indicated that for female faces, the overestimation by the lover was significantly higher than her own overestimation, *t*(29) = 3.93, *p* < .001; for male faces, however, there was no significant difference between him and his lover, *t*(29) = 1.91, *p* = .07.

*(2) Results of rating scores of the exaggeratedly attractive face*

*(i) Results rated by oneself*

Paired-samples *t* tests were conducted to compare the attractiveness scores of one’s own face with the scores of the exaggerated face. The results indicated that for both female face, *t*(29) = −5.65, *p* < .001, and male face, *t*(29) = −3.51, *p* < .01, the rating score of the exaggerated face was much higher than the score of one’s own face. These results indicated that the positive bias in self-face attractiveness evaluation did not surpass the exaggerated faces in couples from the perspective of rating scores.

*(ii) Results rated by one’s lover*

Paired-samples *t* tests were conducted to compare the attractiveness scores of one’s own face with the scores of the exaggerated face rated by one’s lover. The results indicated that for female faces, the rating score of the exaggerated face was much higher than the score of the partner’s face, *t*(29) = −3.48, *p* < .01; however, for male faces, there was no significant difference between the exaggerated face and the partner’s face, *t*(29) = −0.16, *p* = .87.

#### Correlation results of probability evaluation and rating scores

To investigate the consistency of the two indexes of attractiveness evaluation, that is, probability evaluation and rating scores, a correlation analysis was conducted. The results indicated that the correlation between the overestimation probability and the overestimation score, that each image was selected as more attractive by oneself than by others of the same sex (i.e. ‘Self – ssOthers’), reached significance (*r* = .36, *p* < .01). In addition, the correlation between the overestimation probability and the overestimation score that each image was selected as more attractive by one’s lover than by others of the opposite sex (i.e. ‘Lover – osOthers’), also reached significance (*r* = .25, *p* < .01).

## Experiment 2: The Evaluation of Self-Face Attractiveness in Single Individuals

Experiment 2 recruited single individuals as participants to investigate the characteristics of the evaluation of self-face attractiveness. The results of Experiment 2 were compared with those of Experiment 1 to reveal the special features of self-face evaluation among individuals in romantic relationships.

### Methods

#### Participants

A total of 60 single college students (30 females, mean age 20.5 ± 2.0 years with a range of 18–26 years) participated in this experiment for monetary compensation. All participants were required to complete the Chinese adaptation of the ECR (Li & Kato, 2006) to ensure that they were single and not in a romantic relationship. The other recruitment criteria and procedures were identical to those for Experiment 1.

#### Materials

The images of the faces of single subjects were created as described in Experiment 1. The exaggerated faces used in Experiment 2 were the same as those of Experiment 1.

#### Procedure

The formal experimental procedure was identical to the procedure of the first day of Experiment 1. In this experiment, female and male subjects only participated in the evaluation of same-sex faces. Because the subjects were single and had no lovers, it was not necessary to ask them to evaluate the opposite-sex faces, which were adopted to compare with the lovers’ faces in Experiment 1.

#### Data analysis

The data analysis of Experiment 2 was identical to that of Experiment 1, except that there was no ‘Lover’ condition or related ‘osOthers’ (others of the opposite sex) condition in Experiment 2.

### Results

#### Results of probability evaluation

*(1) Results of probability evaluation of one’s own face being selected as more attractive when paired with other same-sex faces (Self vs. Other)*

A Wilcoxon rank-sum test indicated that for both female (*Z* = −4.39, *p* < .001) and male (*Z* = −4.78, *p* < .001) faces, the probability of self-face selection as more attractive (i.e. the *Self* condition in Table 1) was significantly higher than the probability of selection by others of the same sex (i.e. the *ssOthers* condition in Table 1). This result verified the positive bias in the evaluation of self-face attractiveness in single participants from the perspective of probability evaluation.

*(2) Results of probability evaluation of self-face selection as more attractive when paired with the same-sex exaggeratedly attractive face (Self vs. Exaggerated)*

A Wilcoxon rank-sum test was conducted to compare the mean probability of selecting one’s own face as more attractive when paired with other faces of the same sex (‘Self vs. Other’) with that when paired with the same-sex exaggerated face (Self vs. Exaggerated). The results indicated that for both female (*Z* = −4.69, *p* < .001) and male (*Z* = −4.58, *p* < .001) faces, the probability of selecting one’s own face as more attractive in the ‘Self vs. Other’ condition was significantly higher than in the ‘Self vs. Exaggerated’ condition (see Table 1).

To determine whether the participants would think that their own faces were more attractive than the exaggerated face, a one-sample *t* test was conducted with a random probability of 50%. The results indicated that for both the female, *t*(29) = −5.64, *p* < .001, and male, *t*(29) = −3.25, *p* < .01, faces, the probability of evaluating self-face attractiveness as higher than the exaggerated face was significantly lower than 50%. These results indicated that the positive bias in self-face attractiveness evaluation did not surpass the exaggerated faces in single participants from the perspective of probability evaluation.

#### Results of rating scores

*(1) Results of rating scores of self-face attractiveness*

Paired-samples *t* tests indicated that for both female face, *t*(29) = 3.69, *p* < .01, and male face, *t*(29) = 3.78, *p* < .01, one’s own attractiveness score (i.e. the *Self* condition in Table 2) was significantly higher than the score by others of the same sex (i.e. the *ssOthers* condition in Table 2). This result also verified the positive bias in the evaluation of self-face attractiveness in single participants from the perspective of rating score.

To investigate whether there was a gender difference in the overestimation of the attractiveness of one’s own face, we calculated the rating score differences between oneself and others of the same sex (i.e. ‘Self – ssOthers’) for both female (*M* = 0.7, *SD* = 1.1) and male (*M* = 1.3, *SD* = 1.9) faces. An independent-samples *t* test indicated no significant gender difference, *t*(58) = −1.46, *p* = .15.

*(2) Results of rating scores of the exaggeratedly attractive face*

Paired-samples *t* tests were conducted to compare the attractiveness scores of one’s own face and the exaggerated face. The results indicated that for both female face, *t*(29) = −6.83, *p* < .001, and male face, *t*(29) = −3.69, *p* < .001, the rating score of the exaggerated face was much higher than the score of one’s own face. These results indicated that the positive bias in self-face attractiveness evaluation did not surpass the exaggerated faces in single participants from the perspective of rating score.

#### Correlation results of probability evaluation and rating scores

To investigate the consistency of the two indexes of evaluating attractiveness in Experiment 2, that is, probability evaluation and rating scores, a correlation analysis was conducted. The results indicated that the correlation between the overestimation probability and the overestimation score, that each image was selected as more attractive by oneself than by others of the same sex (i.e. ‘Self – ssOthers’), reached significance (*r* = .41, *p* < .01).

#### Comparison of self-face evaluation between couples and single participants

**Figure 1.**
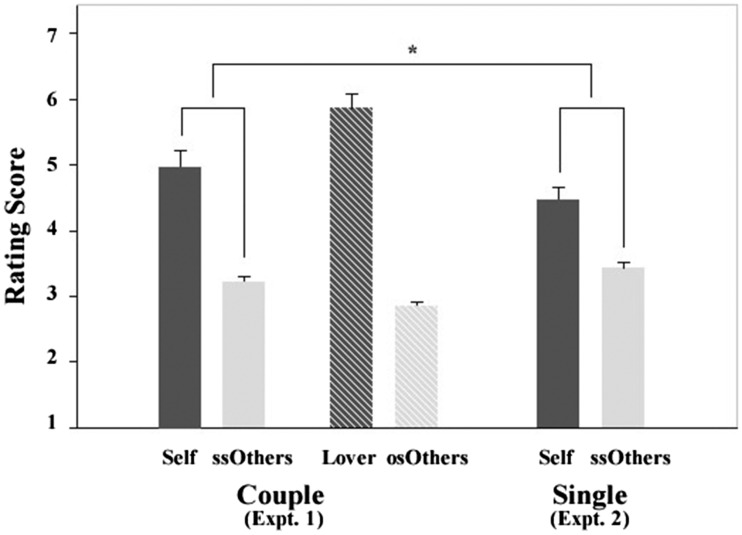
Illustration of rating score of self-face attractiveness by different types of raters. Raters in Experiment 1 were couples involved in a romantic relationship, whereas raters in Experiment 2 were single. ssOthers = same-sex other raters; osOthers = opposite-sex other raters. Error bars indicate standard errors. *p < .05.

A 2 (Subject type: couple, single) × 2 (Evaluator: self, ssOther) two-factor mixed analysis of variance was also conducted on the rating scores of self-face attractiveness, with ‘subject type’ as a between-subjects factor and ‘evaluator’ as a within-subjects factor. The results indicated a significant main effect of evaluator, *F*(1, 118) = 73.30, *p* < .001, and a significant interaction effect between subject type and evaluator, *F*(1, 118) = 4.74, *p* < .05. However, the main effect of subject type did not approach significance, *F*(1, 118) = 0.73, *p* = .40. Post hoc analysis indicated that the score overestimation of one’s own face (i.e. subtracting the rating score by others of the same sex from one’s own score: ‘self – ssOthers’) was significantly higher in couple subjects than that in single subjects, *t*(118) = −2.18, *p* < .05 (see Figure 1). These findings indicated that couple subjects appeared to have a stronger overestimation tendency for self-face attractiveness than single subjects.

To investigate whether there was a similar tendency between the difference of probability overestimation and rating score overestimation between couple subjects (in Experiment 1) and single subjects (in Experiment 2), a correlation analysis was conducted. The results indicated that the correlation between the difference in probability overestimation in couple and single subjects (probability: [*Self – ssOthers*] in Experiment 1 – [*Self – ssOthers*] in Experiment 2) and the difference in score overestimation in couple and single subjects (score: [*Self* – *ssOthers*] in Experiment 1 – [*Self* – *ssOthers*] in Experiment 2) reached significance (*r* = .27, *p* < .05).

## Discussion

Self-evaluation of facial attractiveness plays an important role in interpersonal communication. The current study investigated the influence of a romantic relationship on the self-face attractiveness evaluation in two experiments, using couples (Experiment 1) and single individuals (Experiment 2) as participants. As predicted, the results of the two experiments indicated that the romantic relationship enhanced the positive bias in the evaluation of self-face attractiveness, that is, couple participants indicated a stronger positive bias than did single individuals. In addition, we observed that a person in a romantic relationship was prone to overestimating the attractiveness of his or her lover’s face. However, the two types of overestimation did not surpass the evaluations of the exaggeratedly attractive face.

Experiment 1 found that the probability of the couple participants evaluating one’s own face as more attractive was significantly higher than that by the same-sex others, and they rated their own attractiveness as significantly higher than did others of the same sex. In addition, the probability overestimation and score overestimation exhibited a significant correlation. Experiment 2 replicated the results of Experiment 1. These results consistently verified the positive bias in the evaluation of self-face attractiveness in couples and single participants, utilizing the probability evaluation and the relatively subjective rating method. Our results further demonstrated a positive bias in the evaluation of self-face attractiveness in previous studies (Cai, et al., 2018; Springer et al., 2012; Stillman & Fristina, 2010) and extended the effect to individuals who were part of a couple.

Most importantly, the present study found that individuals in couple status more strongly overestimated self-face attractiveness than did single people. Such findings were directly demonstrated by the rating score comparison between the two experiments. Moreover, the probability overestimation and the rating score overestimation shared a similar tendency, which was supported by the significant correlation between the difference in probability overestimation and score overestimation in couples and single subjects. The stronger positive bias in the evaluation of self-face attractiveness in couples may be because of the higher self-esteem resulting from couple status (Bylsma et al., 1997), which may increase the evaluation of the attractiveness of one’s own face (Bale, 2010). People in couple status have a tendency to imagine an ideal romantic relationship, turning their lovers into more ideal mates, which is similar to the positive illusion of lovers (Murray, Holmes, Bellavia, Griffin, & Dolderman, 2002; Murray, Holmes, & Griffin, 1996a, 1996b). Such expectations may in turn push them to become better and thus overestimate their attractiveness to a greater extent. This assumption was indirectly supported by the fact that those with a higher self-evaluation were more likely to succeed in an intimate relationship (Landolt, Lalumière, & Quinsey, 1995). Moreover, the awareness of self-evaluation affects individual satisfaction with and the development of the relationship (Franzoi, Davis, & Young, 1985; Solano, Batten, & Parish, 1982; Swami & Fyrnham, 2008); some insecure individuals are unable to position themselves and the relationship correctly (Derrick & Murray, 2007; Downey & Feldman, 1996; Murray, Holmes, Griffin, Bellavia, & Rose, 2001; Tucker & Anders, 1999). Nevertheless, the reasons why people in couples (compared with single people) have a stronger positive bias when evaluating self-face attractiveness require further exploration.

Another interesting finding in our study was that people in romantic relationships (compared with others) were prone to overestimating the attractiveness of their lovers’ faces, which was demonstrated by both the probability evaluation and the subjective rating method. When heterosexual interactions become a stable romantic relationship, people’s evaluations of their lovers’ facial attractiveness also change. Price and Dabbs (1974) observed that a girl considered a boy more attractive when she fell in love with him, according to the adage, ‘Beauty is in the eye of the beholder’. Conversely, the attractiveness of other opposite-sex people decreases in their eyes (Johnson & Rusbult, 1989; Miller & Simpson, 1990; Simpson, 1990). However, the aforementioned effect was defined to describe the positive bias favouring the evaluation of the lover’s face over other people’s faces. Our study did not compare the target faces being evaluated but compared the evaluators and identified a similar effect: Someone’s lover (contrary with others) overestimated the partner’s facial attractiveness. Thus, the present study extended the ‘Beauty is in the eye of the beholder’ effect to the comparison between the lover and others as evaluators.

The two results of the present study, that individuals in couples have a stronger overestimation of self-face attractiveness than single people and that people in romantic relationships (compared with others) were prone to overestimating the attractiveness of their lovers’ faces, both argued against the owner hypothesis, which emphasizes the influence of the characteristics of the owner of a face (such as averageness, symmetry and sexual dimorphism) on facial attractiveness (Little & Perrett, 2002). According to the owner hypothesis, the attractiveness evaluation of the same face by different people would exhibit no difference, which did not occur in our study. Instead, our results supported the observer hypothesis, which regards the biological factors, psychological factors and social/cultural factors of observers to be influential factors of facial attractiveness evaluation (Kou et al., 2013).

Our study also found a gender difference in the attractiveness overestimation of one’s own face. Although the male and female subjects both rated their own faces with higher scores than others of the same sex, the overestimation was more significant in males than in females. It is worth mentioning that such a gender difference only occurred in subjects who were in couples, not in single subjects. This difference may have arisen because males have a greater need for esteem for their social role orientation (Kling, Hyde, Showers, & Buswell, 1999), which indicates that males have a stronger self-approval tendency to improve self-face attractiveness evaluation.

In summary, the present study examined the influence of the romantic relationship on the evaluation of self-face attractiveness and found that individuals in couples demonstrated a stronger positive bias in the evaluation of self-face attractiveness than did single people. The findings indicated that the influence of the close relationship is mutual: It affects the evaluation of not only the lover’s facial attractiveness but also the self-face attractiveness. Our results have important implications for the research of self-face evaluation: A close relationship should be considered from the perspective of the evaluator.
